# Elevated non-high-density lipoprotein cholesterol corresponds to a high risk of nephrolithiasis in children

**DOI:** 10.1186/s12894-020-00691-6

**Published:** 2020-08-10

**Authors:** Meiyuan Chen, Jing Xiao, Yuan Du, Miaomiao Wang, Jimeng Ruan, Ye Tian

**Affiliations:** Department of Urology, Beijing Friendship Hospital Affiliated to the Capital Medical University, No. 95, Yong’an Road, Xicheng District, Beijing, 100050 China

**Keywords:** Child, Dyslipidemias, Non-HDL cholesterol, Urolithiasis

## Abstract

**Background:**

Dyslipidemia contributes to the development of nephrolithiasis in adults; however its relationship to urolithiasis in children remains debatable, and will be clarified in the present work.

**Methods:**

A case–control study was performed involving 58 pediatric patients diagnosed with upper urinary tract stones as well as 351 controls. Age, gender, body mass index (BMI), serum calcium, serum uric acid, blood glucose, blood lipids, and compositions of stones were compared.

**Results:**

According to the univariate analysis, uric acid was higher (*P* < 0.01) but serum calcium lower in the stone group than the control (*P* < 0.05). As for the blood lipids, non-high-density lipoprotein cholesterol (non-HDL-c) was significantly higher in the stone group as compared to the control (*P* < 0.01), while total cholesterol, triglycerides, high-density lipoprotein cholesterol, and low-density lipoprotein cholesterol did not show statistical difference between the two groups. In the multivariate analysis, only non-HDL-c and serum uric acid were increased in the stone group (*P* = 0.003 and *P* = 0.008). In the stone compositions’ analysis, serum uric acid and non-HDL-c were associated with percentage of uric acid and pure calcium oxalate stones, respectively.

**Conclusion:**

Non-high-density lipoprotein cholesterol may act as a lipid risk factor for urolithiasis in children.

## Background

The incidence of pediatric stones and the hospitalization rate are increasing yearly [1, 2]. Routh et al. reported that the number of pediatric urolithiasis cases per 100,000 hospitalized patients was 18.4 in 1999 and 57.0 in 2008, with an 10.6% adjusted annual growth rate [3] . Moreover, a 4% per year increase rate of pediatric kidney stones was documented in a 25-year population-based study [4].

Mechanisms underlying pediatric urolithiasis remain unclear. However, urinary metabolic abnormalities, including hyperoxaluria, hypocitraturia, hypercalcinuria, and hyperuricosuria are considered to play a role in the process [5]. Furthermore, a study of adults showed the total cholesterol (TG), triglycerides (TC), and low-density lipoprotein cholesterol (LDL-c) were higher, but the high-density lipoprotein cholesterol (HDL-c) was lower in calculus patients than healthy persons, supporting the theory that dyslipidemia contributes to the formation of urinary stones [6]. A similar result has been reported in several other studies [7–9]. Conversely, in a study on non-stone children, TC, LDL, and TG were negatively, but HDL positively correlated with urinary citrate events in obese participants [10].

Therefore, we hypothesized that the abnormal lipid metabolism may be involved in nephrolithiasis among children. Hence, in the present work, we examined the lipid profile of pediatric patients diagnosed with upper urinary tract stones and analyzed the association between dyslipidemia and stone formation in children.

## Methods

### Subjects

This case-control study included 409 children aged 3–18 years who visited Beijing Friendship Hospital Affiliated to Capital Medical University of China from January 2016 to December 2017. The stone group consisted of 58 children who received surgery after an initial diagnosis of kidney or ureteral stones according to ultrasonography, X-ray, or CT examinations. The control group consisted of 351 children who were admitted to the hospital for diseases or conditions other than urolithiasis, including inguinal hernia, orthodontics, and deaf children awaiting cochlear implants. All control group participants denied a urinary calculi history and underwent ultrasound examinations to exclude it. Patients with congenital or acquired urinary system malformations or obstructions, endocrine-related stones, or who were taking antihyperlipidemic drugs or diet were excluded from this study. All cases were double-checked by the research team. This study was approved by the Ethics Committee of Beijing Friendship Hospital Affiliated to the Capital Medical University (2019-P2–183-01). Written informed consent was obtained from the children’s parent(s) or guardian included in the study.

### Methods

After an overnight fast, the venous blood samples (6–8 ml) were drawn from all the participants in the morning between 8:00 am and 9:00 am. BMI, serum calcium, blood glucose, serum uric acid, TC, TG, HDL-c, LDL-c, and non-HDL cholesterol (non-HDL-c) were measured. Non-HDL-c was obtained by subtracting the HDL-c from TC [11]. The childhood obesity and body mass index Z-score (BMI-Z score) were defined according to previous studies [12, 13]. Dyslipidemia was identified referring to the following criterion: hypercholesterolemia (TC ≥ 5.2 mmol/L), hypertriglyceridemia (TG ≥ 1.76 mmol/L), high LDL-cholesterolemia (LDL-c ≥ 3.38 mmol/L), and low HDL-cholesterolemia (HDL-c ≤ 1.04 mmol/L) [14].

### Analysis of stone composition

A Fourier infrared spectrometer (LIIR-20, Lambda Scientific Instrument, Tianjin, China) was used to analyze the stone composition. The stones were washed with water and dried in an oven at 70–100 °C. Approximately 1 mg of the stone sample powder was mixed with 200 mg of pure potassium bromide which had been sufficiently dried beforehand, and then grounded in an agate mortar to a size of 2 μm or less. The mixture was baked for 10 to 30 min, and then taken out and pressed by a tableting machine to prepare a translucent sheet, which was then quickly scanned in an infrared spectrum tank. The computer automatically parsed and reported the stone components after drawing the spectrum. The phase that reached at least 50% of the total calculus composition was considered to be the major component of the stone.

### Statistical analysis

All data were presented as the percentages or means ± standard deviation (SD). Blood lipids were shown as dichotomous or continuous variables. All the statistical analyses were conducted using SPSS v21.0 software (SPSS Inc., Chicago, IL). The Mann–Whitney U-test or Chi-squared test was used for comparisons between the two groups. Logistic regression was applied to estimate the independent contributions of the serum composition to the formation of stone. Correlations between stone composition and UA or non-HDL-c were made using the Spearman test. A *P* value of < 0.05 was considered statistically significant.

## Results

A total of 409 subjects were involved in the study, with 58 in the stone group and 351 in the control group. All the subjects were of Han Chinese ethnicity. In the stone group, there were 32 (55.2%) cases of unilateral ureteral calculi, 17 (29.3%) cases of unilateral kidney stones, 3 (5.2%) cases of bilateral kidney stones, 1 (1.9%) case of bilateral ureteral stones, 4 (6.9%) cases of unilateral kidney stones with unilateral ureteral calculi, and 1 (1.9%) case of bilateral kidney stones with unilateral ureteral stone. The anthropometric data of the two groups are shown in Table 1. There were no statistical differences in age, height, and weight between the two groups.

**Table 1:** Anthropometrics and lipid profile of the stone and control groups

Variables	Stone group (***n*** = 58)	Control group (***n*** = 351)	***P*** value
Age (year)	7.92 ± 3.63	8.36 ± 4.20	0.727^a^
Height (m)	1.30 ± 0.22	1.32 ± 0.25	0.855^a^
Weight (kg)	32.23 ± 17.27	34.25 ± 18.85	0.649^a^
BMI-Z score	0.25 ± 2.07	0.64 ± 1.42	0.392^a^
Calcium (mmol/L)	2.43 ± 0.11	2.47 ± 0.12	0.003^a^
Blood sugar (mmol/L)	4.89 ± 0.53	4.87 ± 0.50	0.834^a^
Uric acid (umol/L)	327.24 ± 95.35	286.24 ± 73.44	0.002^a^
TC (mmol/L)	4.38 ± 0.78	4.17 ± 0.74	0.066^a^
TG (mmol/L)	1.01 ± 0.51	0.91 ± 0.45	0.281^a^
HDL-c (mmol/L)	1.36 ± 0.34	1.30 ± 0.27	0.328^a^
LDL-c (mmol/L)	2.32 ± 0.45	2.42 ± 0.57	0.268^a^
Non-HDL-c (mmol/L)	3.05 ± 0.60	2.55 ± 1.09	0.004^a^
Gender, n (%)			0.456^b^
Male	41 (70.7)	228 (65.0)	
Female	17 (29.3)	123 (35.0)	
Overweight, n (%)	20 (34.5)	130 (37.0)	0.770^b^
Dyslipidemia status, n (%)			
Hypercholesterolemia	5 (11.6)	22 (7.0)	0.350^b^
Hypertriglyceridemia	4 (9.3)	19 (6.1)	0.502^b^
High LDL-c	1 (2.4)	13 (4.2)	1.000^b^
Low HDL-c	7 (16.7)	50 (16.0)	0.827^b^

*BMI* body mass index, *TC* total cholesterol, *TG* triglyceride, *HDL-c* high-density lipoprotein cholesterol, *LDL-c* low-density lipoprotein cholesterol, *non-HDL-c* non-HDL cholesterol

*P*-values are obtained from ^a^Mann–Whitney U-test and ^b^Fisher’s exact test

Data are represented as means ± standard deviation (SD) unless otherwise indicated

Serum calcium and uric acid levels were higher in the stone group than the control (*p* = 0.003 and *p* = 0.002); however, there was no significant difference in BMI or blood glucose between the two groups. In the case of blood lipids, TC, TG, HDL-c and LDL-c did not show statistical difference, but non-HDL-c was significantly enhanced in the stone group (*P* = 0.004) relative to control. Calcium, uric acid, TC, and non-HDL-c were identified by the univariate analyses as potential risk factors for urinary stones (Table 2). In the multivariate analysis, uric acid and non-HDL-c were the only two independent risk factors for stone formation (*p* = 0.008 and *p* = 0.003) (Table 3).

**Table 2:** Univariate logistic regression analysis of risk factors for urinary stones

Variables	***P***-value	OR	95% CI
Age	0.449	0.973	0.908–1.044
Gender	0.395	0.769	0.419–1.409
BMI	0.433	0.972	0.906–1.043
Calcium	0.012	0.062	0.007–0.548
Blood sugar	0.763	1.088	0.630–1.878
Uric acid	0.001	1.006	1.002–1.009
TC	0.089	1.412	0.948–2.102
TG	0.208	1.500	0.798–2.817
HDL-c	0.258	1.911	0.622–5.867
LDL-c	0.276	0.707	0.379–1.319
Non-HDL-c	0.004	1.728	1.194–2.501

*CI* confidence interval, *OR* odds ratio

**Table 3:** Multivariate logistic regression analysis of risk factors for urinary stones

Variables	B	***P*** value	OR	95%CI
Calcium	−1.327	0.306	0.265	0.021–3.368
Uric acid	0.005	0.008	1.005	1.001–1.009
Non-HDL-c	0.587	0.003	1.798	1.226–2.636

Stone compositions’ analysis revealed that the majority of stones were mainly composed of calcium oxalate (CaOx) (76%) and the rest contained mainly uric acid (UA), cystine (ST), and calcium phosphate (CaP). Among CaOx-dominated stones, 61% were mixed with CaP or UA, 61% were sorted as calcium oxalate monohydrate (COM), and 39% were calcium oxalate dihydrate (COD) (Table 4). The correlation analysis showed that formation of UA stones were positively related to serum UA level (*r* = 0.546), while CaOx stones had a reverse and weak relationship with UA (*r* = − 0.300). Only pure CaOx stones were positively related to serum non-HDL-c level (*r* = 0.521). The ST stones correlated with neither UA nor non-HDL-c (Table 5).

**Table 4:** Analysis of patients’ stone compositions

Stone composition	Number of patients
CaOx (total)	44
CaOx+CaP	22
COM + Cap	13
COD+Cap	9
CaOx+UA	5
COM + UA	3
COD+UA	2
CaOx	17
COM	11
COD	6
UA(total)	7
UA + CaOx	1
UA	6
ST	6
CaP+CaOx	1

*CaOx* calcium oxalate, *COM* calcium oxalate monohydrate, *COD* calcium oxalate dihydrate, *CaP* calcium phosphate, *UA* uric acid, *ST* cystine stones

**Table 5:** Correlation between stone compositions and serum UA or non-HDL-c

	CaOx (total)	ST	UA (total)	CaOx
Serum UA	−0.300*	−0.130	0.546**	−0.149
Non-HDL-c	−0.092	−0.078	0.119	0.521**

CaOx (total) refers to stones whose main component is CaOx; CaOx refers to pure CaOx stones

*P < 0.05 **P < 0.001

## Discussion

The prevalence of urinary stones in children is rising, and their pathogenesis has still not been fully elucidated [2]. Several studies indicated that the lipid metabolism abnormalities may contribute to the elevation of urine oxalate in adult calculus patients [6–8]. However, the relevance of lipid disorder to the stone formation in children is unclear. Non-HDL-c signifies cholesterol contained in lipoproteins except for HDL, which includes LDL, intermediate-density lipoprotein (IDL), very-low-density lipoprotein (VLDL), and chylomicron, thus covering nearly all established atherogenic lipid particles.

Fasting is unnecessary for the detection of non-HDL-c [15]. The present work demonstrated a possible association between non-HDL-c and risk for development of urolithiasis in children, which, as far as we know, has never been reported before. Moreover, the stone analysis showed just a correlation between non-HDL-c and pure CaOx stones present. Although this finding has not yet proved that non-HDL-c mainly affects the formation of CaOx stones, it may suggest that children with calcium oxalate stones need to pay more attention to the existence of abnormal lipid metabolism.

Our findings could be explained by many reasons. First, non-HDL-c is well known as a risk factor of atherosclerosis as well as vascular calcification. Kidney is a vascular dominant organ, which might be prone to non-HDL-c elicited damage and manifests itself as stones. Based on the viewpoint that the vasa recta arises from efferent arterioles of the juxtamedullary glomeruli, which is vulnerable to hypoxia and hypertonia around the renal medulla and sensitive to blood flow change in the U-shaped counter-current multiplier system [16], vasa recta might be most likely subject to the non-HDL-c related injuries. Yencilek et al. found early lesions of atherosclerosis and impairment of systemic endothelial cell function in patients with stones [17]. So the vascular calcification may be the initial event of hydroxyapatite formation in the renal papilla or the origin of Randall plaque (RP) [18].

Second, from a pathological point of view, urolithiasis could be a kind of ectopic calcification and RP the original step of stone formation. Khan et al. using renal nipple biopsy found that spherulitic CaP crystals deposited in the interstitium as well as in the laminated basement membrane of the tubular epithelia in patients with CaOx kidney stones. So it was inferred that the formation of RPs was an ectopic calcification process, similar to vascular calcification [19]. Considering the involvement of non-HDL-c in vascular calcification, it may also contribute to the formation of kidney stones. A nanoscale study indicated the RPs present at both the tip of the Henle loop and the vasa recta [20]. So, although the two structures are adjacent to each other, given that the non-HDL-c could not pass through the glomerular filtration, it only affects the formation of kidney stones in the vasa recta.

Moreover, a retrospective clinical study showed that administration of statins prevented stone development in hyperlipidemia patients, and this effect persisted even with other factors adjusted [21]. This result has been conformed in another clinical study and the author speculated the protective behavior of statins may be related to anti-inflammatory, anti-oxidative, and anti-atherosclerotic features of the medicines [22]. All the above evidence indirectly suggests that the abnormal lipid metabolism might be responsible for the stone formation in children. However, it should be mentioned that non-HDL cholesterol is not a “solo” cholesterol, as mentioned above, which includes a variety of cholesterol components mainly carried by LDL and VLDL. In this study, LDL failed to show its significance as an independent risk factor by multivariate analysis, so it is reasonable to speculate that VLDL cholesterol may play a role in this relationship. In fact, VLDL cholesterol was found to be an independent risk factor in a cohort study of coronary heart disease (CHD). Population-attributable risk proportion (PAR%) of CHD risk associated with VLDL cholesterol alone was higher than that of CHD risk associated with LDL cholesterol alone. About three-quarters of patients at-risk of CHD (who had elevated VLDL cholesterol) present with normal LDL cholesterol levels [23]. Considering the relationship between CHD and atherosclerosis, this may explain why non-HDL-c is an independent risk factor rather than LDL-c in our study.

In the present study, stone compositions’ analyses revealed that most children had CaOx stones, among which 61% were verified as COM and 39% were COD (Table 4). This result agrees with previous reports showing the main type of CaOx stone would be COM in both adults and children of China [24]. However, as there is an apparent regional variation on the features of urolithiasis, other areas would show different situations [25]. Another finding of the present study (i.e. blood calcium acted not as a predictor of urinary stones) might be inconsistent with previous reports demonstrating COD is related to hyperoxalaturia and COM to hypercalciuria [26]. But there are also studies indicating CaOx stones are multi-etiological; only 11% of children with stones develop hypercalciuria [27], and hypercalciuria is not a risk factor for stones [28]. The noticeable discrepancy and complexity of this issue warrants further research for clarification.

Being the end product of purine metabolism, UA is mainly cleared through renal excretion. Hyperuricemia might cause hyperuricuria, which acts as one of the three contributors of UA stones thus explaining the association between elevated serum UA and formation of UA stones found in our study. This result is also consistent with a previous study by Lim et al. [29] showing a correlation between serum UA and UA stone in adult patients. However, our finding also found a weakly negative correlation between CaOx stones with serum UA, which might be due to the high proportion of the CaOx stones among all the cases.

There are some limitations in our research. First, the sample size was small, which may reduce the potential to detect risk factors of disease. Also for this cause, the contributors to each type of urolithiasis in children could not be unveiled. Second, the lack of urine metabolism analysis caused incomplete exploration of the mechanisms. Third, without genetic testing conducted, the gene-derived urolithiasis could not be screened out. Finally, this study only revealed the correlation between urolithiasis and non-HDL-c in children, but the causal link between them could not be verified.

## Conclusions

As elevated non-HDL-c and uric acid in serum is closely correlated to urolithiasis in children, it might become a new target for the prevention and treatment of the disease.

## Data Availability

The datasets used and/or analyzed during the current study are available from the corresponding author on reasonable request.

## Abbreviations

BMI: Body mass index
TC: Total cholesterol
TG: Triglyceride
HDL-c: High-density lipoprotein cholesterol
LDL-c: Low-density lipoprotein cholesterol
Non-HDL-c: Non-HDL cholesterol
IDL: Intermediate-density lipoprotein
VLDL: Very-low-density lipoprotein
CI: Confidence interval
OR: Odds ratio
CaOx: Calcium oxalate
CaP: Calcium phosphate
UA: Uric acid
ST: Cystine stones
RP: Randall plaque
